# Challenges in Diagnosis and Management of Infantile Deep Neck Abscess: A Case Report and Literature Review

**DOI:** 10.7759/cureus.55543

**Published:** 2024-03-05

**Authors:** Heba K Alkoheji, Wasan M Alharbi, Fatima N Abulfateh, Mai Nasser, Mohamed Alshehabi

**Affiliations:** 1 Otolaryngology - Head and Neck Surgery, The Royal Medical Services, Riffa, BHR

**Keywords:** management, diagnosis, abscess, deep neck, infantile

## Abstract

A deep neck abscess is a relatively rare bacterial infection in infants that can rapidly progress to life-threatening complications. Mostly, the patients present with fever and neck pain. Some may present with dysphonia, sore throat, torticollis, trismus, or neck swelling. Early diagnosis and management can prevent life-threatening complications, such as airway obstruction, mediastinitis, and tracheitis. In this report, we present a case of a six-month-old infant presenting with retropharyngeal and parapharyngeal abscesses with prompt diagnosis leading to complete recovery of the patient.

## Introduction

A deep neck abscess is an uncommon condition in infants defined as a collection of pus in the deep cervical space. This severe condition can rapidly progress to life-threatening complications such as acute mediastinitis, septic thrombophlebitis, and respiratory failure. Upper respiratory tract infection is the most common cause of deep neck infections (DNIs) in children, followed by odontogenic origin. The spread of the infection via direct continuity or lymphatic node drainage from oropharyngeal infections to retropharyngeal lymph nodes is the proposed mechanism of the infection [1-3].

DNIs can be located in spaces above the level of the hyoid bone (peritonsillar, submandibular, parapharyngeal, buccal, parotid, masticatory/temporal spaces, and Ludwig's angina), along the length of the neck (retropharyngeal, danger, prevertebral, and carotid spaces), and below the level of the hyoid bone (anterior visceral or pretracheal space) [2]. The most common site of DNIs identified in children, as described by a retrospective study, is parapharyngeal space, seen in 42.3% of the cases, followed by submandibular, retropharyngeal, and peritonsillar spaces [4].

The most common pathogen identified in children was *Staphylococcus aureus*, predominantly methicillin-sensitive *Staphylococcus aureus*. Coagulase-negative *Staphylococcus*, *Mycobacterium tuberculosis*, anaerobic bacteria, *Viridians streptococci*, and *Klebsiella pneumonia* were among the other pathogens isolated. Infants’ clinical presentation is deceiving and non-specific, which makes diagnosis challenging. Children usually present with neck mass, swelling, fever, sore throat, dyspnea, and limited neck motion [4].

The diagnosis of deep neck abscess in children is challenging as children tend to have non-specific signs and symptoms and oropharyngeal examination is difficult to be conducted due to its size. Early diagnosis and management can prevent life-threatening complications, such as airway obstruction, mediastinitis, and tracheitis. Approximately 11.6% have been found to develop complications in a retrospective study in children [4-6].

In this report, we present a case of a six-month-old infant presenting with retropharyngeal and parapharyngeal abscesses, with prompt diagnosis leading to complete recovery of the patient. The uniqueness of this case report stems from the condition's rarity in infancy, particularly with the presence of retropharyngeal and parapharyngeal abscesses. For instance, those aged two to six years are more prone to retropharyngeal abscess due to the more prominent lymphatic nodal systems, including nodes of Rouvière that tend to be less prominent in younger children. Older children are also less likely to develop these abscesses due to regression of the nodes by four years of age. Furthermore, the presentation in infants is unusual, with clinical symptoms that may be deceptive. We also discovered a scarcity of case reports or research on deep neck abscesses in our region. Fortunately, early detection and treatment result in complete infant recovery with favorable outcomes in less than six days from presentation [7].

## Case presentation

We present a case of a six-month-old female infant with no documented alerts, who presented to the Bahrain Defense Force Hospital emergency department with a right deep neck abscess.

A previously well six-month-old female infant was brought to our emergency department by her mother with a two-day history of inability to turn her head to the right side associated with excessive drooling of mucus, and a two-week history of cough and runny nose. The patient had a one-day history of subjective fever but no history of poor oral intake, muffled voice, preceding trauma, stridor, or known foreign body ingestion. The patient presented to another hospital for bronchiolitis two weeks ago, was managed with supportive treatment, and was discharged. On general examination, the patient was vitally stable and afebrile but hypoactive with a weak cry. The child had torticollis, and her neck showed a right-sided swelling below the angle of the mandible around 3 x 3 cm with no overlying skin changes. On palpation, there was tenderness on touch and multiple palpable cervical lymph nodes. A throat examination revealed a posterior pharyngeal wall swelling toward the right side with a pooling of purulent material, but no identifiable sinus or uvular deviation was noted. The ear examination was unremarkable. Furthermore, the patient was examined using a flexible fiberoptic laryngoscope, which showed bulging of the posterior pharyngeal wall toward the right side with significant airway narrowing. On admission, the patient had leukocytosis (WBC count: 27.41 x 10^9^/L) with predominant neutrophilia (67.2%) and elevated inflammatory markers (C-reactive protein (CRP): 49.9 mg/l).

Ultrasound (US) of the neck was performed, revealing loculated turbid fluid collection measuring 4 x 3 cm with a thick wall seen on the right side of the neck, features suggestive of neck abscess with multiple enlarged cervical lymph nodes. Abscess aspirate revealed group A beta-hemolytic *Streptococci*, while acid-fast bacilli stain and culture for tuberculosis were negative. A blood culture was collected and revealed negative results with no pathogens identified. A lateral neck X-ray was obtained, which showed prevertebral soft tissue thickening with significant narrowing of the airway.

**Figure 1:**
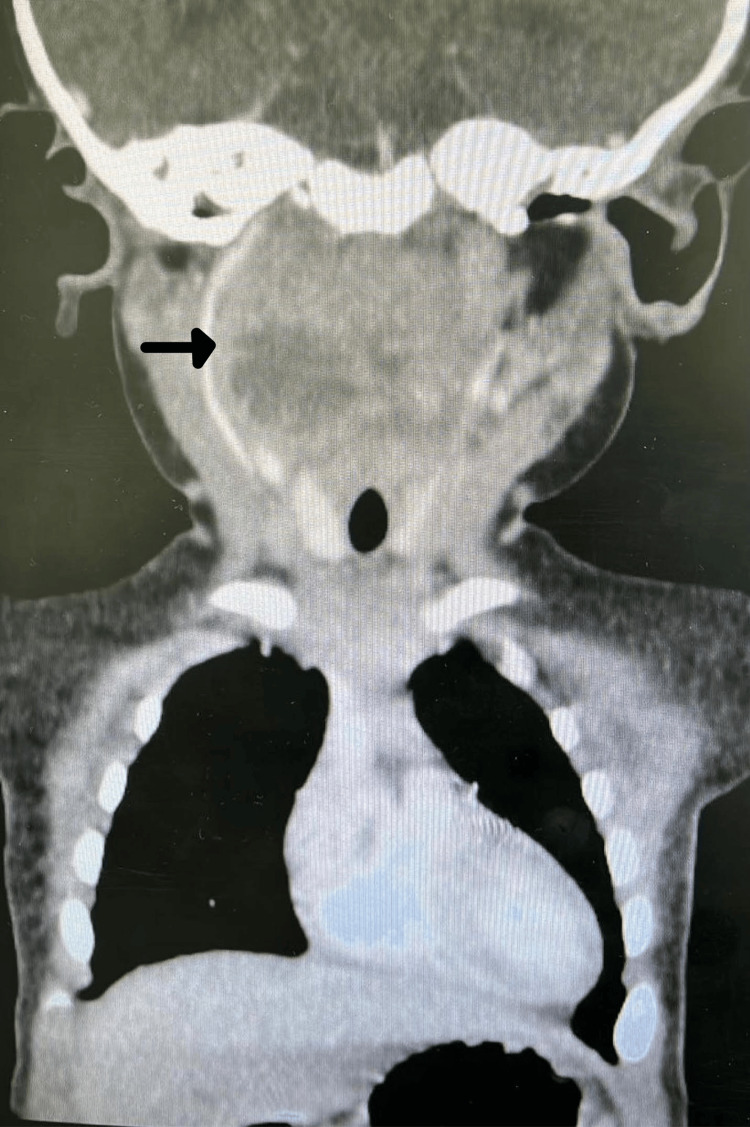
Coronal view of CT scan showing right-sided retropharyngeal and parapharyngeal abscess measuring about 27 x 33 x 12 mm in maximum dimensions that connects to the right side of oropharynx at C3 level with marked compromise of airway and multiple enlarged lymph nodes. CT: computed tomography; C3: cervical spine 3.

**Figure 2:**
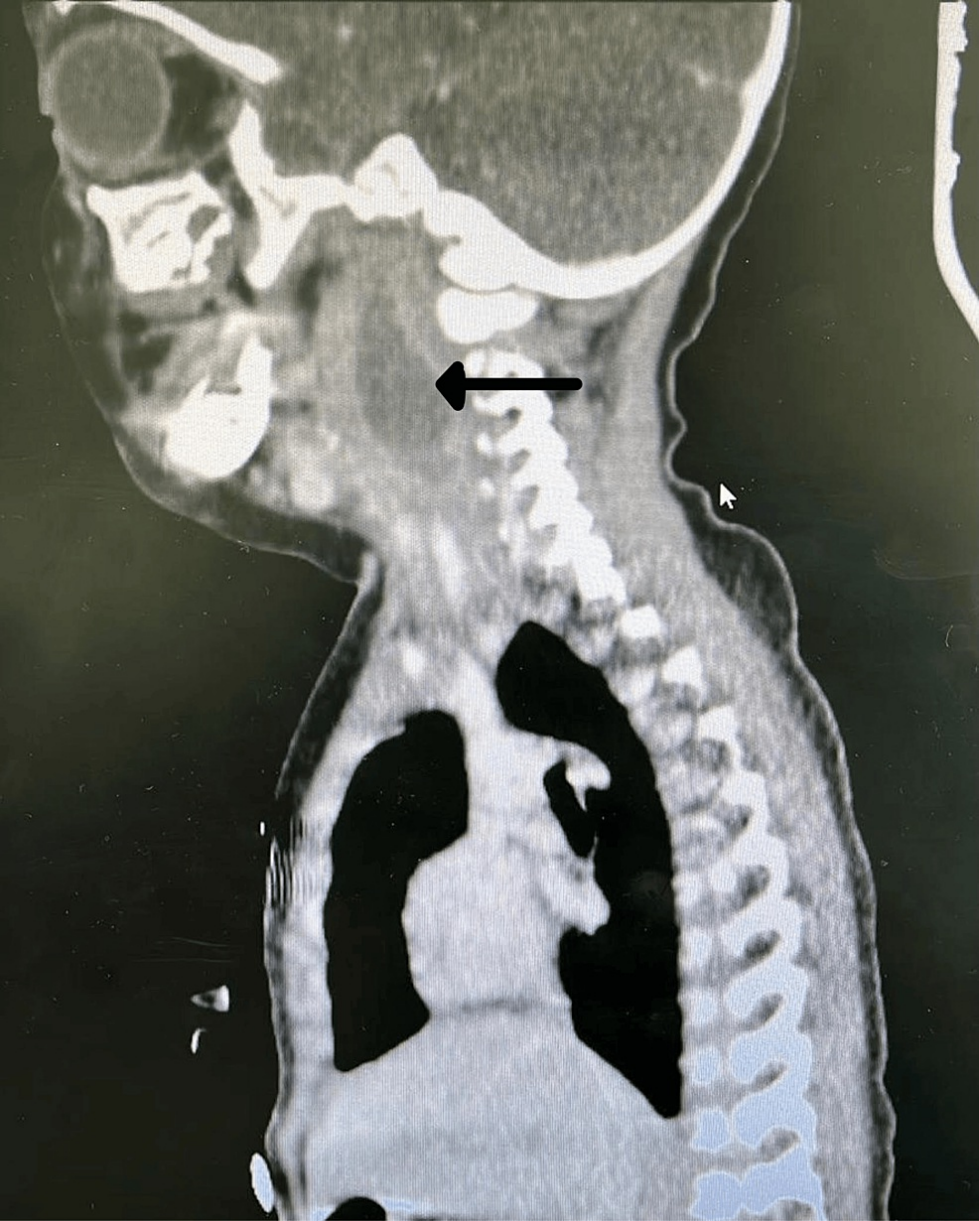
Axial view of CT scan showing right-sided retropharyngeal and parapharyngeal abscess measuring about 27 x 33 x 12 mm in maximum dimensions that connects to the right side of oropharynx at C3 level with marked compromise of airway and multiple enlarged lymph nodes. CT: computed tomography; C3: cervical spine 3.

Initially, the patient was started on clindamycin 90 mg three times a day and vancomycin 60 mg four times a day empirically. She also underwent ultrasound-guided aspiration of pus under local anesthesia. On day two, the patient's swelling of the neck and drooling of saliva improved. However, she developed stridor and had a low-grade fever of 38°C; thus, contrast-enhanced computed tomography (CT) scan of the neck was then ordered, revealing a right-sided retropharyngeal and parapharyngeal abscess measuring about 27 x 33 x 12 mm in maximum dimensions that connects to the right side of oropharynx at cervical spine 3 (C3) level with marked compromise of airway and multiple enlarged lymph nodes (Figures 1, 2). Therefore, emergency trans-oral retropharyngeal abscess drainage was done on the same day. Intraoperatively, there was a prominent bulge on the posterior pharyngeal wall at the right side extending above the glottic area filled with a large amount of pus. The patient was shifted with an endotracheal tube to the pediatric intensive care unit afterward and clindamycin and vancomycin were continued. The patient was then discharged home on day five with cefdinir 40 mg once daily for two weeks after she showed clinical and laboratory improvement with lateral neck X-ray post-extubation showing a considerable reduction in retropharyngeal space expansion.

The baby was doing well on follow-up after one and two weeks with no fever or sore throat episodes. On examination, the throat was clear, with no swellings.

## Discussion

The incidence of deep neck abscesses in infants is not explicitly stated in the literature. However, it is widely acknowledged that deep neck abscesses in infants are uncommon but can result in critical or life-threatening situations. A Japanese study found a mean incidence of 4.0 ± 1.9 cases per year in pediatric patients [8]. A retrospective analysis study was conducted in India. Over a 15-year period, 510 children under the age of five with deep neck abscesses were identified. The average age of diagnosis was 23.6 months, with *Staphylococcus aureus* being the most common pathogen. For instance, those with retropharyngeal or multiple abscesses are more likely to develop complications [9]. Furthermore, parapharyngeal abscesses have been identified with an average age of 3.3 years according to a five-year retrospective study [3].

According to a study conducted in the United States to determine the incidence of retropharyngeal abscess in children, the incidence increased from 2.98 per 100,000 to 4.10 per 100,000 in 2012. It was highest in children under the age of five, particularly in boys. The study, however, did not mention the incidence in infants [10].

It should be noted that these figures are specific to the study settings and may not reflect the prevalence in other populations or regions. No study described the prevalence of deep neck abscesses in children in the Middle East, indicating the need for more research on this condition.

Clinical presentation is subtle in infants, making diagnosis challenging. However, the presence of a neck mass or swelling should raise suspicion of DNIs and should be included in the differential diagnosis. X-ray of the neck may identify a retropharyngeal abscess if the prevertebral soft tissue shadow is greater than 7 mm at the C2 (cervical spine 2) level or more than 14 mm at the C6 (cervical spine 6) level. A contrast-enhanced CT scan is the gold standard imaging because it can accurately identify the presence and anatomical location of the abscess, allowing for more precise surgical planning [1].

The differential diagnosis of this disease widely depends on the clinical presentation of the infant. Stridor should warrant ruling out conditions like acute epiglottitis, bacterial tracheitis, and croup. Neck mass in an infant by itself can have a list of differentials. It is important to consider congenital causes such as brachial cleft cysts, dermoid cysts, hemangiomas, and thyroglossal duct cysts in an infant. The presence of fever can narrow the differentials into superimposed infection on congenital masses, inflammatory neck masses such as reactive lymphadenopathy, infectious lymphadenitis, or Kawasaki disease. Malignant neoplasia such as lymphoma, nasopharyngeal carcinoma, and rhabdomyosarcoma are rare but possible etiologies. The presence of acquired torticollis should necessitate the ruling out of life-threatening conditions, such as retropharyngeal abscess, suppurative jugular thrombophlebitis, cervical spine injury, spine epidural hematoma, and central nervous system tumor. Other common causes include acute infections with compensatory muscle spasms such as upper respiratory tract infection and cervical adenitis. The list, however, is not exhaustive [1,11,12].

Deep neck abscesses in children require a combination of medical and surgical treatment. Empiric antibiotics should be started; the antibiotic of choice has yet to be determined in clinical trials. Intravenous nafcillin or vancomycin plus gentamycin or tobramycin, ampicillin/sulbactam, or clindamycin, on the other hand, are widely used. If the patient has methicillin-resistant *Staphylococcus aureus* (MRSA) and is not responding to initial empiric antibiotics, a vancomycin/linezolid + cefepime regimen can be used. Antibiotics are typically given for three weeks or longer, depending on the presence of complications [1].

There are no specific treatment guidelines for deep neck abscesses in children. The recommendation, however, is dependent on the severity of the illness and the type of organism causing the abscess. As a result, in patients with retropharyngeal abscesses who exhibit signs of severe airway compromise such as stridor, anxiety, or retractions, emergency surgical drainage is indicated. For those with mature abscesses (2.5 cm2), surgical drainage is also recommended. Those who do not have an airway compromise may be given a trial of antibiotic therapy for 24-48 hours without surgical drainage, especially if the abscess is not large and mature. Another factor to consider is the failure of antibiotic therapy and the development of a large abscess (2.5 cm2), which necessitates surgical drainage. However, there is disagreement about the surgical management of either retropharyngeal or parapharyngeal abscesses, with some clinicians advocating early intervention and others advocating conservative treatment. Those without surgical drainage had contradictory results, with some studies reporting a high-resolution rate of 75% and others reporting a low-resolution rate of 31.5% success. Early surgical drainage (transoral approach) was performed with good clinical outcomes in a retrospective study of parapharyngeal abscesses in children [4,13-15].

There are no guidelines or studies that compare the external versus transoral approach in children under general anesthesia. However, research and case studies frequently show the transoral approach. The transoral approach was also revealed to be associated with lower risks and is less invasive. An external approach, on the other hand, is preferred if there is severe trismus or the abscess is adjacent to large vessels [16].

We discovered a few case studies on the proposed topic while reviewing the literature (Table 1). For example, after presenting with fever, left neck swelling, diarrhea, and dysphagia, a four-month-old girl was successfully diagnosed with a parapharyngeal abscess (via US and contrast-enhanced CT scan). The child also had hypersalivation and ptosis of the left eyelid. The patient was diagnosed with a parapharyngeal abscess and was discharged on the eighth day after undergoing general anesthesia for a left tonsillectomy with incision and drainage. Clindamycin and piperacillin/tazobactam were given in combination, and the patient was discharged with significant improvement [16].

**Table 1:** Summary of deep neck abscesses in infants from the literature. US: ultrasound.

Year	Age	Presentation	Time from symptoms to diagnosis	Diagnostic modality	Management	Outcome
2022 [16]	4 months old	Fever, left-sided neck swelling, diarrhea, dysphagia, ptosis of the left eye	No duration mentioned	Contrast CT scan: left parapharyngeal abscess (37 × 26 × 32 mm)	Left tonsillectomy, incision, and evacuation of the abscess (transoral approach) were done. The patient was started on clindamycin and piperacillin/tazobactam	Discharged on the 8th postoperative day
2021 [17]	14 months old	High-grade fever, neck stiffness, restriction of neck movements, decreased oral intake, dysphagia, muffled sounds, obstructive sleep apnea	Twelve days	Neck X-ray: widened cervical soft tissue in the prevertebral space. Ultrasound: retropharyngeal space fluid collection. Contrast CT scan: retropharyngeal septated fluid collection (8 × 3 × 6 cm) extending over the length of the cervical spine	Emergency incision and drainage under general anesthesia were performed. The patient was covered with clindamycin and ceftriaxone	Discharged on the 10th day after admission
2020 [18]	3 months old	Inspiratory stridor, difficulty breathing when taking feeds, nasal discharge	No mention of duration. The patient had symptoms for three days, was initially managed as croup, and due to non-improvement, a CT scan was done in which diagnosis was established	Neck X‑ray: Thickened prevertebral shadow (12 mm at C3 level) and airway narrowing. US and contrast CT scan: retropharyngeal abscess with dimensions of 1.7 × 1 × 2.1 cm extending up to pyriform sinus	Urgent intraoral transpharyngeal incision and drainage were performed. The antibiotics of choice were ceftriaxone and vancomycin.	Repeated non-contrast CT scan was negative. No mention of when the patient was discharged
2017 [19]	4 months old	Fever, lethargy, left lateral neck swelling, stridor, poor oral intake	Four days of symptoms and was diagnosed on presentation	Contrasted CT scan: collection in the parapharyngeal and retropharyngeal spaces with diameters of 5.3 × 8 cm that extend to the skull base and the left sternoclavicular junction	Emergency intraoral incision and drainage were done. The patient was started on cefotaxime and metronidazole	Discharged home after one week
2009 [20]	9 months old	Torticollis	Six weeks	X-ray of the neck: enlarged retropharyngeal and posterior tracheal spaces. US of the neck: cervical lymphadenopathy. Contrast-enhanced CT scan: left posterior pharynx swelling and left acute mastoiditis	Medical therapy only: ceftriaxone and flucloxacillin	No mention of when the patient was discharged. The patient underwent a CT scan after four weeks showing complete resolution

Alkhadem et al. presented a 14-month-old female patient with high-grade fever, neck stiffness, restriction of neck movements, decreased oral intake, dysphagia, muffled quality of voice, and obstructive sleep apnea for seven days in a recent case report. The patient's lab results showed increased inflammatory markers, as expected. The initial US of the neck revealed an extensive fluid collection in the retropharyngeal space. The patient had a large retropharyngeal abscess from acute tonsillitis and a peritonsillar abscess, which led to mediastinitis, according to a contrast CT scan. Due to the patient's unstable condition, an emergency incision and drainage were performed under general anesthesia. The patient was then started on empirical intravenous (IV) ceftriaxone and clindamycin for 10 days before being discharged on oral co-amoxiclav for *Staphylococcus aureus*-positive pus [17].

Another case report was performed on a retropharyngeal abscess in a three-month-old male who presented with stridor, difficulty breathing, and nasal discharge for three days. The patient was suspected of having croup, but due to unresolved disease despite IV ceftriaxone and nebulization, a contrast CT scan was performed, which revealed an abscess in the retropharyngeal area. Furthermore, on the advice of radiologists, a US scan was performed because some collections may be isodense on CT. Due to the presence of stridor, urgent incision and drainage under general anesthesia were preferred over aspiration. Ceftriaxone IV antibiotics were continued with the addition of vancomycin. A Gram-stained drained smear revealed MRSA, the patient's symptoms improved, and a non-contrast CT scan revealed no new collection one week later [18].

Balasubramanian et al. also discovered a severe, extensive deep neck abscess in a previously healthy four-month-old female infant. The patient had a four-day history of lethargy, fever, and swelling of the left neck, as well as a two-day history of poor oral intake and stridor. The patient was immediately intubated, and a contrast CT scan revealed a massive left parapharyngeal and retropharyngeal abscess causing airway narrowing. The patient was treated with an emergency surgical intraoral incision and drainage with endoscopic assistance, and antibiotics (IV cefotaxime and metronidazole) were administered for one week, with complete recovery [19].

Mydam et al. described an exciting path of retropharyngeal abscess spread in a nine-month-old girl. The patient had torticollis for six weeks and had no other symptoms. Following a thorough examination, it was discovered that the patient had a retropharyngeal abscess caused by left acute mastoiditis. For seven days, the patient was given IV antibiotics (IV ceftriaxone and flucloxacillin), followed by seven days of oral antibiotics. Physiotherapy was started for seven days, which resulted in complete resolution on a CT scan after four weeks with no need for surgical management [20].

## Conclusions

Deep neck abscesses are a rare occurrence in infants, and the condition can lead to serious complications, such as respiratory failure, with a rate of complications of 11.6%. Prompt diagnosis and treatment are required to protect the child from potentially fatal outcomes, with treatment encompassing both surgical and medical approaches. The exact prevalence in our region is unknown. As a result, additional epidemiological studies are required to accurately determine the incidence and prevalence, which can vary greatly depending on geographical location, demographics, and healthcare access. This case report identified a case in the Middle East in the Kingdom of Bahrain that resulted in successful medical and surgical approaches, serving as a model for future cases with the same complaint.
